# Mini-Mental State Examination as a Predictor of Mortality among Older People Referred to Secondary Mental Healthcare

**DOI:** 10.1371/journal.pone.0105312

**Published:** 2014-09-03

**Authors:** Yu-Ping Su, Chin-Kuo Chang, Richard D. Hayes, Gayan Perera, Matthew Broadbent, David To, Matthew Hotopf, Robert Stewart

**Affiliations:** 1 King's College London (Institute of Psychiatry), London, United Kingdom; 2 Dept of Psychiatry, Cathay General Hospital, Taipei, Taiwan; 3 School of Medicine, Fu-Jen Catholic University, Taipei, Taiwan; 4 South London and Maudsley NHS Foundation Trust, London, United Kingdom; University Medical Center Rotterdam, Netherlands

## Abstract

**Background:**

Lower levels of cognitive function have been found to be associated with higher mortality in older people, particularly in dementia, but the association in people with other mental disorders is still inconclusive.

**Methods and Findings:**

Data were analysed from a large mental health case register serving a geographic catchment of 1.23 million residents, and associations were investigated between cognitive function measured by the Mini-Mental State Examination (MMSE) and survival in patients aged 65 years old and over. Cox regressions were carried out, adjusting for age, gender, psychiatric diagnosis, ethnicity, marital status, and area-level socioeconomic index. A total of 6,704 subjects were involved, including 3,368 of them having a dementia diagnosis and 3,336 of them with depression or other diagnoses. Descriptive outcomes by Kaplan-Meier curves showed significant differences between those with normal and impaired cognitive function (MMSE score<25), regardless of a dementia diagnosis. As a whole, the group with lower cognitive function had an adjusted hazard ratio (HR) of 1.42 (95% CI: 1.28, 1.58) regardless of diagnosis. An HR of 1.23 (95% CI: 1.18, 1.28) per quintile increment of MMSE was also estimated after confounding control. A linear trend of MMSE in quintiles was observed for the subgroups of dementia and other non-dementia diagnoses (both p-values<0.001). However, a threshold effect of MMSE score under 20 was found for the specific diagnosis subgroups of depression.

**Conclusion:**

Current study identified an association between cognitive impairment and increased mortality in older people using secondary mental health services regardless of a dementia diagnosis. Causal pathways between this exposure and outcome (for example, suboptimal healthcare) need further investigation.

## Introduction

Lower cognitive function in dementia is a predictor of mortality [1]–[3], although this has primarily been described in severe impairment, and effects of milder dysfunction remain controversial [4]–[10]. Lower cognitive function in older people without dementia has also been found to be associated with higher mortality, although this again remains inconclusive [8], [11]–[17] and evidence on interventions to prevent mortality remains limited [18]. A better understanding is therefore needed of factors influencing prognosis in older people with and without dementia to aid care planning and clinical decision making [8], [18], [19].

Depression is commonly comorbid with dementia, and associated itself with worse outcome [10], [16], [20], although the relationship between the two may be complex, with depression potentially a cause of dementia, a consequence, a prodromal symptom, and/or a condition with shared risk factors [20], [21]. Some research has suggested that depression is an independent risk factor for mortality in people without dementia [10], [22], although others have not found this [16], and the diagnosis of depressive disorder itself is recognised to be associated with elevated mortality risk particularly in older people [23].

In the study described here, we analysed data from a retrospective cohort aged 65 years and above, using information from a large secondary mental healthcare provider in southeast London. We hypothesised that lower cognitive function assessed by Mini-Mental State Examination (MMSE) would be an independent risk factor for mortality in those with dementia, depression and those with a psychiatric diagnosis other than the former ones.

## Methods

### Study setting

The South London and Maudsley NHS Foundation Trust (SLAM) Case Register has been described in detail previously [24]. In brief, the Clinical Record Interactive Search (CRIS) program allows researchers to access full but anonymised data from a large electronic mental health records dataset [25]. Within the UK National Health Service, secondary mental health care is provided according to defined geographic catchment areas. SLAM is one of the largest mental health providers in Europe, delivering comprehensive secondary mental health services to a population of approximately 1.23 million residents in four London boroughs (Lewisham, Lambeth, Southwark, and Croydon), including outpatient/community, inpatient, and general hospital liaison services. Currently, there are records on over 220,000 cases accessed by CRIS and this database has been extensively utilised [26]–[28]. The SLAM Case Register has been approved as an anonymised data resource for secondary analyses by Oxfordshire Research Ethics Committee C (08/H0606/71+5) and governance is provided for all projects by a patient-led oversight committee.

### Analysed sample

All cases with at least one MMSE score recorded during the period between 1^st^ Jan 2007 and 31^st^ Dec 2010 were first identified. This sample was restricted to cases aged at least 65 years old at the date of this MMSE record, and excluded those with a recorded delirium diagnosis (ICD-10 code: F05) within three months before or after the date of the MMSE assessment. MMSE scores recorded during routine clinical care were derived from dedicated structured fields on the electronic health record, supplemented by a specific information extraction application developed using Generalised Architecture for Text Engineering (GATE) software: a natural language processing architecture which takes into account the linguistic context of a word or phrase of interest, thus allowing structured data to be obtained from open-text fields. The specific GATE applications were developed by programmers and validated against human raters to extract and code MMSE scores and associated dates of assessment with a recall (sensitivity) of 97% and precision (positive predictive value) of 98%. For the purpose of this analysis, only MMSE scores with denominators >25 were included, and scores were excluded if two different numerators were assigned to the same date.

### Covariates and analysis subgroups

Mental disorder diagnoses are categorised in structured fields on the source clinical record according to World Health Organization International Classification of Diseases 10th edition (ICD-10) codes. In addition, a further GATE information extraction application identifies text strings associated with a diagnosis statement in correspondence fields, and this was used for additional searches on predefined diagnostic terms. The following groups were defined for analysis: 1) a group with dementia was defined on the basis of a diagnosis (ICD-10 codes F00–F03) recorded any time before or up to six months after the index MMSE assessment; 2) within the non-dementia group, a group with depression (F32–F33) anytime before or up to six months after the MMSE assessment was specified for analysis; 3) the remainder within the non-dementia group consisted of elders with other diagnoses before the MMSE assessment, including schizophrenia and related psychotic disorders (F20–F29), anxiety spectrum and stress-related disorders (F40–F48), bipolar affective disorder (F30–F31), and others. Demographic data included age (defined at the index MMSE) and gender. Ethnic group was classified from a structured field in the record as: i) white British and other white background; ii) African, Caribbean and other black background; iii) east Asian; iv) south Asian; and v) mixed, unknown, or others. Marital status was categorised from a structured field into: i) married, civil partner, or cohabiting; ii) single; iii) separated or divorced; iv) widowed; and v) unknown. Area-level socioeconomic status was estimated from an index of multiple deprivation applied to the UK Census lower super output area (standard geographic areas with an average 1,500 residents). This index is defined by seven domains assessed in the national Census: employment, income, education, health, barriers to housing and services, crime and the living environment. Indices were calculated from 2001 Census output and were divided by tertiles for this analysis.

### Mortality outcome

The outcome of interest in this analysis was all-cause mortality occurring from January 2007 to the end of July 2011. Information about each death was collected through a nationwide mortality tracing linked to the SLAM database on a monthly routine basis. In UK, all death certifications are linked by NHS number (a unique identifier for each UK NHS service user) to all healthcare providers, keeping these records up to date.

### Statistical analysis and ethical approval

The follow-up period was defined as the duration from date of the first MMSE assessment in the observation period to the date of death or the end of follow up (31^st^ Jul 2011). The sample was first described by covariates and absolute risks of mortality during the follow-up period. Cox proportional hazard models were then used to investigate covariate associations with and without age and gender adjustment. MMSE as an exposure was categorised in two ways: i) a binary variable comparing impairment (0–24) against non-impairment (25–30); ii) five groups of reducing scores defined by quintiles in the whole analysed sample (N = 6,704). Kaplan–Meier curves were used to compare mortality between 0–24 and 25–30 categories of MMSE scores in groups with and without a dementia diagnosis. Further analyses of associations between mortality and MMSE score in descending quintile groups were then performed for the following three diagnostic categories specified above: 1) dementia; 2) depression, no dementia; and 3) other psychiatric diagnoses, neither dementia nor depression.

## Results

**Figure 1:**
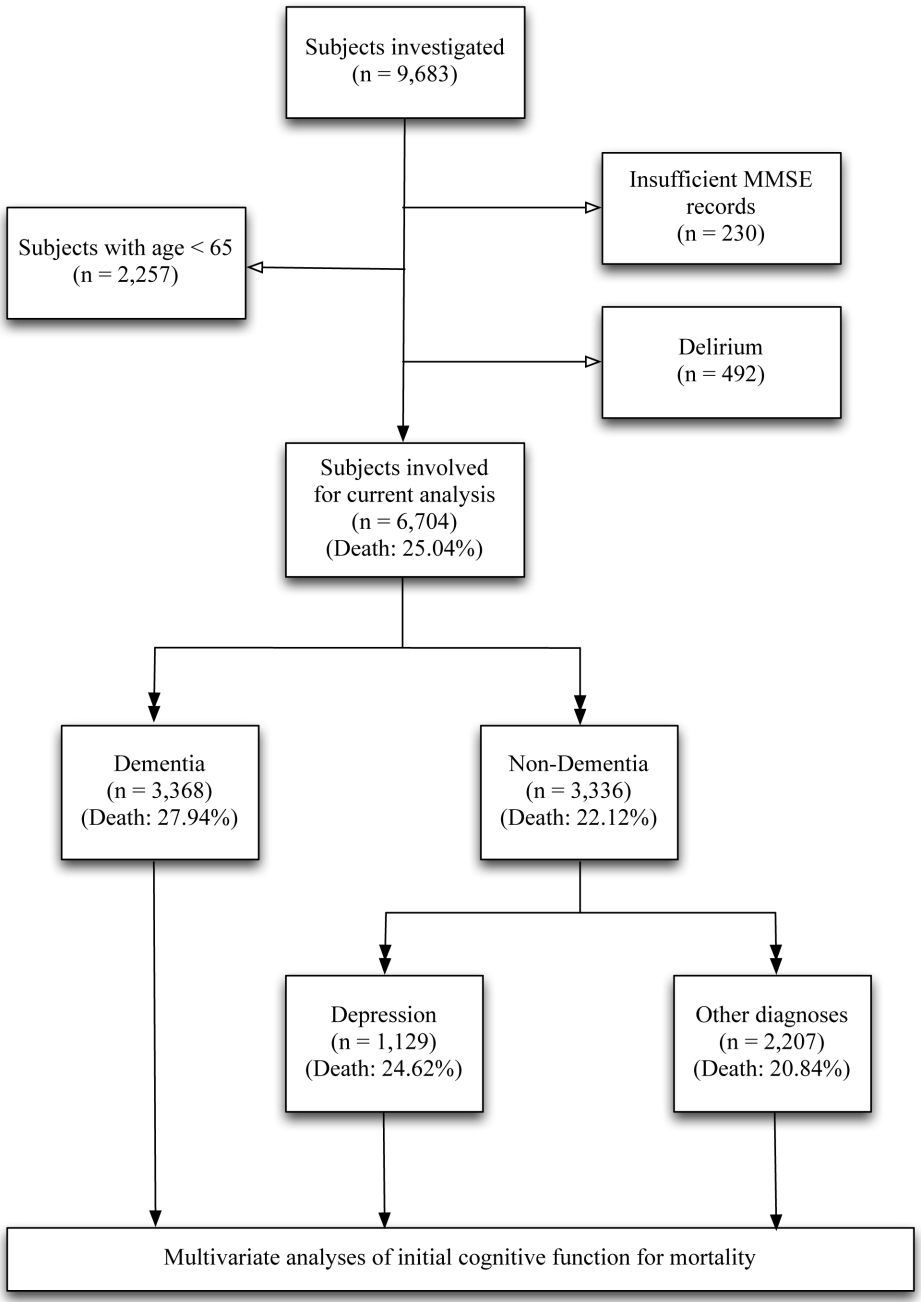
Diagram of sample selection and diagnosis subgroups.

A total of 9,683 subjects were identified with at least one MMSE score during the period of 01/01/2007 to 31/12/2010. Of these, 230 with an MMSE denominator less than 25, 2,257 aged less than 65 years old, and 492 with diagnoses of delirium were excluded (Figure 1). Of the remaining 6,704 subjects in the analysed sample, the mean (SD) index MMSE score was 21.2 (6.6), and 1,679 (25.0%) died prior to the end of the follow-up (31/07/2011). Around half (n = 3,368; 50.2%) had a diagnosis of dementia, and in those without dementia (n = 3,336), depression was the most common primary diagnosis (n = 1,129; 33.8%) followed by 30.8% with schizophrenia and related psychotic disorders, 17.1% with anxiety spectrum and stress-related disorders, and 6.8% with bipolar affective disorder.

**Figure 2:**
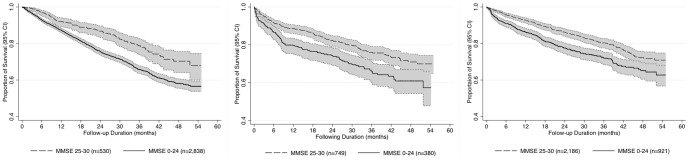
Kaplan–Meier curves comparing groups with and without cognitive impairment (MMSE<25) among subjects with dementia (left, n = 3,336), depression (middle, n = 1,129), and other diagnoses (right, n = 2,207). Footnote: Shaded areas present 95% confidence intervals; all the p-values for log-rank tests <0.01.

The mean (SD) follow-up period was 26.5 (14.8) months. Figure 2 illustrates Kaplan-Meier survival curves comparing groups with and without cognitive impairment stratified by diagnosis subgroups (dementia, depression, and others). In all three subgroups, the difference between MMSE groups was statistically significant on log-rank tests (p-value<0.001) with similar patterns.

**Figure 3:**
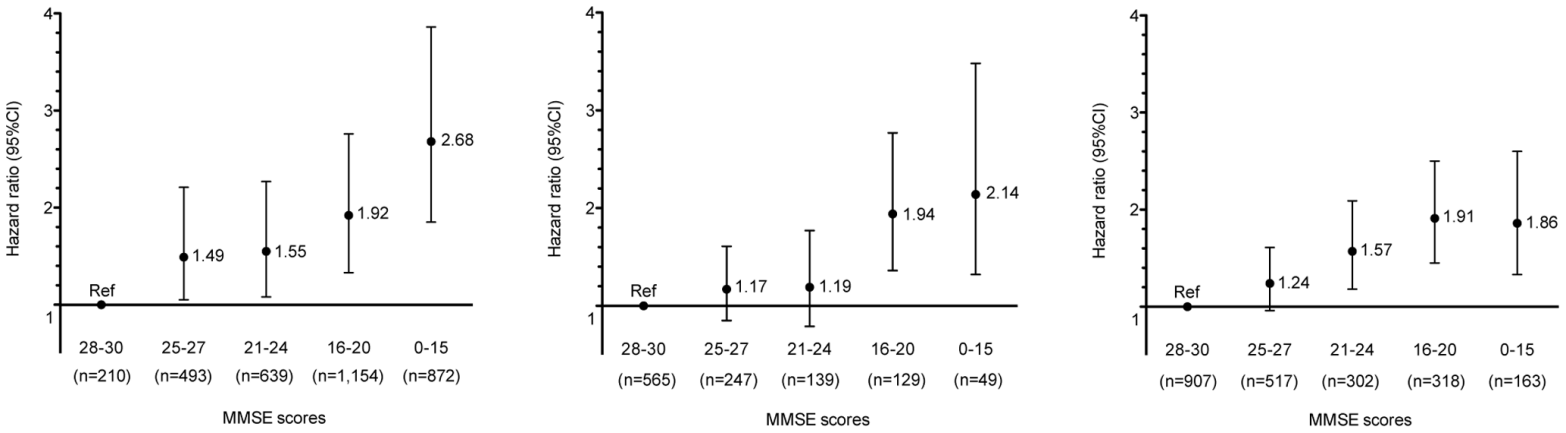
Adjusted associations Mini-Mental State Examination score and mortality in subjects with dementia (left, n = 3,368), depression (middle, n = 1,129), and other diagnoses (right, n = 2,207). Footnote: All were adjusted for age at assessment, gender, ethnicity group, marital status, and index of deprivation score. Hazard ratio per unit increment for quintiles = 1.26 (95% CI 1.18–1.34; p-value<0.01), 1.21 (95% CI 1.10–1.34; p-value<0.01), and 1.18 (95% CI 1.10–1.27; p-value<0.01).

Table 1 revealed the basic characteristics of the study subjects. In Table 2, the unadjusted analyses showed that older age and male gender were associated with lower survival. After adjustment for age and gender, mortality was significantly raised in those with a diagnosis of depression, but did not differ significantly between those with/without dementia. Mortality risk was significantly lower in black and south Asian compared to white groups, and was higher in single and widowed compared to married/cohabiting subjects. Higher deprivation score was associated with increased risk of mortality with a significant linear trend (p-value<0.001). A fully adjusted hazard ratio (HR) of 1.42 (95% CI: 1.28, 1.58) was identified for MMSE score<25. When MMSE score was divided into quintiles, a significant linear trend was evident for all subjects with a fully adjusted HR of 1.23 (95% CI: 1.18, 1.28) for each unit increment in quintiles of MMSE estimated (Table 2), as well as in those with dementia (Figure 3 left; fully adjusted HR 1.25, 95% CI: 1.18–1.33), without dementia (adjusted HR 1.18, 95% CI: 1.12–1.25) with depression but no dementia (Figure 3 middle; 1.21, 1.10–1.33), and with other non-dementia diagnoses but no depression or dementia (Figure 3 right; 1.19, 1.11–1.28).

**Table 1:** Distribution of baseline covariates among clients of a secondary mental health service provider aged 65 years old or more and by psychiatric diagnoses.

Risk factors	Number (%)/mean ± SD			
	All (N = 6,704)	Dementia (n = 3,368)	Depression (n = 1,129)	Others (n = 2,207)
Age at MMSE assessment	80.24±7.69	82.04±7.00	77.97±7.64	78.64±8.06
Gender				
Female	4,116 (61.4%)	2,111 (62.7%)	717 (63.5%)	1,288 (58.4%)
Male	2,587 (38.6%)	1,256 (37.3%)	412 (36.5%)	919 (41.6%)
Ethnic group				
White	5,341 (79.7%)	2,715 (80.6%)	958 (84.9%)	1,668 (75.6%)
Black	757 (11.3%)	392 (11.6%)	83 (7.4%)	282 (12.8%)
East Asia	96 (1.4%)	41 (1.2%)	18 (1.6%)	37 (1.7%)
South Asia	165 (2.5%)	79 (2.4%)	23 (2.0%)	63 (2.9%)
Unknown/Mixed/Others	345 (5.1%)	141 (4.2%)	47 (4.2%)	157 (7.1%)
Marital status				
Married/Civil partner/Cohabiting	1,970 (29.4%)	1,056 (31.4%)	312 (27.7%)	602 (27.3%)
Single	954 (14.2%)	403 (12.0%)	161 (14.3%)	390 (17.7%)
Separated/Divorced	629 (9.4%)	249 (7.4%)	119 (10.6%)	261 (11.8%)
Widowed	2,530 (37.7%)	1,378 (40.9%)	446 (39.5%)	706 (32.0%)
Unknown	621 (9.3%)	282 (8.4%)	91 (8.1%)	248 (11.2%)
Area-level deprivation score				
1^st^ tertile (1.63–22.16, the lest deprived group)	2,202 (32.8%)	1,143 (34.0%)	329 (29.1%)	730 (33.1%)
2^nd^ tertile (22.17–35.25)	2,137 (31.9%)	1,061 (31.5%)	362 (32.1%)	714 (32.4%)
3^rd^ tertile (35.26–65.53, the most deprived group)	2,167 (32.3%)	1,075 (31.9%)	392 (35.7%)	700 (31.7%)
Missing	198 (3.0%)	89 (2.6%)	46 (4.1%)	63 (2.9%)
Cognitive function				
Non-impairment (MMSE: 30-25)	2,565 (38.3%)	530 (15.7%)	749 (66.3%)	1,286 (58.3%)
Impairment (MMSE: 24-0)	4,139 (61.7%)	2,838 (84.3%)	380 (33.7%)	921 (41.7%)
MMSE score in quintiles				
1^st^ quintile (30-28)	1,259 (18.8%)	131 (3.9%)	445 (39.4%)	683 (31.0%)
2^nd^ quintile (27-25)	1,306 (19.5%)	399 (11.9%)	304 (26.9%)	603 (27.3%)
3^rd^ quintile (24-21)	1,454 (21.7%)	812 (24.1%)	202 (17.9%)	440 (19.9%)
4^th^ quintile (20-16)	1,395 (20.8%)	1,003 (29.8%)	115 (10.2%)	277 (12.6%)
5^th^ quintile (15-0)	1,290 (19.2%)	1,023 (30.4%)	63 (5.6%)	204 (9.2%)

**Table 2:** Effect of baseline covariates and associations with mortality assessed by Cox regressions (N = 6,704).

Risk factors	Death (%)	Hazard Ratio (95% Confidence Interval)	
		Crude	Adjusted
Age at MMSE assessment	–	1.05 (1.05, 1.06)*	–
Gender			
Female	22.79	Ref	–
Male	28.64	1.35 (1.22, 1.48)*	
Ethnic group			
White	27.20	Ref	Ref^a^
Black	15.06	0.52 (0.43, 0.62)*	0.61 (0.50, 0.74)*
East Asia	14.58	0.51 (0.30, 0.86)∧	0.66 (0.39, 1.12)
South Asia	13.94	0.49 (0.33, 0.75)*	0.59 (0.39, 0.90)∧
Unknown/Mixed/Others	21.74	0.87 (0.69, 1.09)	0.94 (0.70, 1.11)
Marital status			
Married/Civil partner/Cohabiting	21.12	Ref	Ref^a^
Single	26.42	1.30 (1.11, 1.52)*	1.33 (1.13, 1.55)*
Separated/Divorced	16.69	0.77 (0.62, 0.95)∧	0.93 (0.75, 1.16)
Widowed	27.47	1.35 (1.20, 1.53)*	1.30 (1.14, 1.48)*
Unknown	33.98	1.59 (1.35, 1.88)*	1.50 (1.27, 1.78)*
Area-level deprivation score			
1^st^ tertile (1.63–22.16, the lest deprived group)	22.84	Ref^#^	Ref^a^^, ^ +
2^nd^ tertile (22.17–35.25)	26.16	1.11 (0.98, 1.25)	1.14 (1.02, 1.29)*
3^rd^ tertile (35.26–65.53, the most deprived group)	26.81	1.20 (1.06, 1.35)*	1.26 (1.11, 1.42)*
Missing	18.18	0.78 (0.55, 1.09)	0.79 (0.66, 1.10)
Diagnosis of dementia			
No	22.12	Ref	Ref^a^
Yes	27.94	1.28 (1.16, 1.41)*	1.08 (0.98, 1.19)
Diagnosis of depression			
No	24.57	Ref	Ref^a^
Yes	26.39	1.11 (0.99, 1.25)	1.32 (1.17, 1.74)*
Cognitive function			
Normal (MMSE: 30-25)	19.10	Ref	Ref^b^
Impaired (MMSE: 24-0)	28.73	1.62 (1.46, 1.80)*	1.42 (1.28, 1.58)*
MMSE score	–	0.96 (0.95, 0.96)*	0.96 (0.96, 0.97)^b^^, ^ *
MMSE score in quintiles			
1^st^ quintile (30-28)	17.39	Ref+	Ref^b^^, ^ +
2^nd^ quintile (27-25)	20.75	1.20 (1.01, 1.44)∧	1.08 (0.90, 1.29)
3^rd^ quintile (24-21)	32.31	1.38 (1.17, 1.64)*	1.23 (1.03, 1.46)∧
4^th^ quintile (20-16)	29.32	1.84 (1.56, 2.17)*	1.54 (1.30, 1.83)*
5^th^ quintile (15-0)	34.19	2.24 (1.90, 2.63)*	1.90 (1.60, 2.25)*

a Age and gender adjusted.

b Adjusted for age at assessment, gender, ethnicity group, marital status, and index of deprivation score; HR = 1.23 (95% CI: 1.18, 1.28) for each quintile increment.

* P-value<0.01,

∧ p-value<0.05.

+ P-value of test for linear trend <0.001.

## Discussion

In total, 6,704 older subjects were included for analyses, with 61.4% for females, 3,368 of them diagnosed as dementia and 3,336 of them as depression or other mental disorders. No matter if a dementia diagnosis was given, people with impaired cognitive function (MMSE score<25) showed worse survival with statistical significance. Linear trends of MMSE in quintiles were found for the groups of dementia and other non-dementia diagnoses. Cognitive impairment has been suggested to be associated with increased mortality in community samples [29]–[32], but this relationship between cognitive impairment and mortality in clinical samples has not been clear. Using a large anonymised electronic database containing mental health records for a geographic catchment of approximately 1.23 million residents, we investigated the relationship between cognitive function and mortality risk in older people within a mean period of over 2 years. We found that MMSE score was a substantial predictor of mortality, regardless of diagnosis. However, although linear trends were found for the quintiles of MMSE scores for different diagnosis subgroups, there still appeared to be individual dose-response patterns in terms of their effects on mortality.

People with dementia are known to have higher mortality rates than general population, and increasing severity of dementia is also associated with increased risk of mortality [33]. As cognitive impairment itself is a core construct in dementia, cognitive impairment is unsurprisingly a predictor of mortality in dementia [1]–[3]. Our analysis supported this, in that lower MMSE scores strongly predicted subsequent mortality in people with a dementia diagnosis (Figure 2 left). However, the effect was noted to be similar in people without a dementia diagnosis (Figure 2 middle & right), as has also been suggested [34]. Several studies have found that people with severe mental disorders (schizophrenia, depression, schizoaffective disorder, and bipolar disorder) have a higher mortality rate and a shortened life expectancy, comparing to the general population [35]–[38], an association which was not mediated by exposure to psychotropic agents [39], although may be exacerbated by severity and chronicity [40]. In our analysis, a clear trend for increasing mortality risk was observed for subjects with lower cognitive function in the absence of a dementia diagnosis, including subgroups with depression and other non-dementia diagnoses (Figure 3 middle & right). Our results provided another probable predictor of mortality for subjected without dementia but with depression or other mental illnesses. Although it is possible that some of these subjects had dementia which was not mentioned in the clinical record and therefore not identified here, it is also possible that lower MMSE score was reflecting a level of severity of the underlying functional disorder – for example, the ‘pseudo dementia’ syndrome in severe depression.

It has been suggested that the association between cognitive dysfunction and mortality reflects an underlying global decline in health affecting cognition and directly causing mortality [12], [41]. Cognitive impairment complicates neurological and cerebrovascular disease, and other chronic medical illness, which are known risk factors for mortality [15], [17]. However, while moderate to severe cognitive impairment has been previously found to be associated with higher mortality [4], [5], [42]–[44], the association with milder impairment is less clear [4], [6], [43]. Although cognitive decline measured longitudinally may better predict of subsequent mortality [6], [41], [45], our study suggests that a single MMSE assessment has a substantial strength of association with risk.

Strengths of the study include the large sample size and the generalisability of the sample to other clinical populations (i.e. defined by clinical rather than research diagnoses and with exposure status measured by a widely used instrument). However, it is important to bear in mind limitations of the data which were drawn directly from routine clinical practice rather than for the purpose of research, resulting in that the information about education level and physical comorbidities was not available for confounding control. The study only analysed the first recorded MMSE for each subject during the observation period, in order to simplify the analytic approach. Beyond cognition, the severity of the underlying mental disorder was not analysed and covariates were limited primarily to sociodemographic factors. Besides, because of a concern for the arbitrary cut-off point of 25 as acceptable denominators of MMSE assessment outcomes (although it only took 2.8% of all the service users older than 65 years old with MMSE scores, n = 196), a set of sensitivity analyses including the ones with a denominator equal or less than 25 revealed very similar outcomes.

Our findings therefore suggest that cognitive dysfunction as measured here is associated with higher risk of mortality in patients seen in routine mental healthcare regardless of the underlying diagnosis. Further research is needed to clarify whether there are underlying disorders associated with lower MMSE whose detection and treatment might improve prognosis – i.e. around the viability of a preventative intervention.
